# Genotoxic Evaluation of Surfactin C in Chinese Hamster Lung Cell Line

**DOI:** 10.5487/TR.2009.25.1.047

**Published:** 2009-03-01

**Authors:** Jong Hwan Lim, In Bae Song, Byung Kwon Park, Myoung Seok Kim, Youn-Hwan Hwang, Hyo In Yun

**Affiliations:** 18 B&C Biopharm, Advanced Institutes of Convergence Technology, 906-5 Iuidong, Yeongtonggu, Suwonsi, Gyeonggido, 443-270 Korea; 28 Institute of Veterinary Animal Science, College of Veterinary Medicine, Chungnam National University, 220, Gungdong, Yuseonggu, Daejeon, 305-764 Korea; 38 JEONJIN BIO, Daegu, 704-230 Korea

**Keywords:** Surfactin C, Chromosome aberration test, MTT test, Chinese Hamster Cell

## Abstract

To investigate the mutation inducibility of surfactin C, we performed the chromosome aberration assay with Chinese hamster lung cells *in vitro*. The colorimetric MTT screening assay was carried out to determine the cytotoxicity index (IC_50_) of surfactin C. The IC_50_ value was 125 μg/ml. For the chromosome aberration test of surfactin C, the maximum concentration was employed as 125 μg/ml, followed by 62.5 and 31.25 μg/ml for the lower concentrations, with or without metabolic activation (S9). Cyclophosphamide and mitomycin C were used as positive controls in the presence and absence of S9 metabolic activation, respectively. These results showed that surfactin C was not capable of inducing chromosome aberration, as measured by the chromosome aberration test using Chinese hamster lung cell line. There is no evidence for surfactin C to have a genotoxic potential.

## INTRODUCTION

Biosurfactants are proteins with detergent, emulsifier, and antimicrobial actions that have potential application in environmental applications such as the treatment of organic pollutants and oil recovery (Georgiou *et al.*, 1990; Desai and Banat, 1997; Banat *et al.*, 2000; Rodrigues *et al.*, 2006). Especially, microbial surfactants have several advantages over synthetic surfactants such as lower toxicity, easier biodegradability, better environmental compatibility, higher selectivity and specific activity at extreme temperatures, pH and salinity (Georgiou *et al.*, 1990; Desai and Banat, 1997; Banat *et al.*, 2000; Rodrigues *et al.*, 2006).

Surfactin showed that it had a strong surface tension-lowering activity and showed antiviral, antitumor, fibrinolytic and hypocholesterolemic activities (Arima *et al.*, 1968; Singh and Cameotra, 2004; Mulligan, 2005). It is a mixture of isofoms which slightly differ in their physiological properties due to a variation in the chain length and branching of its hydroxy fatty acid component as well as substitutions of the amino acid components of the peptide ring (Kanatomo *et al.*, 1995). Among of them, surfactin C (Fig. 1) enhanced endogenous thrombolytic reactions by activation of plasminogen activator and inhibition of platelet aggregation (Kikuch and Hasumi, 2002; Lim *et al.*, 2005). It showed antimicrobial activity against methicillin-resis-

**Fig. 1.** The structure of surfactin C.

tant Staphylococcus aureus (MRSA) and inhibited various inflammatory mediators such as cyclooxygenase-2, interleukin-1β and inducible nitric oxide synthase (Hwang *et al.*, 2005a, b; Takahashi *et al.*, 2006). Moreover, it was less toxic than other surfactants as judged from the results of an acute toxicity study in mice (Pakr *et al.*, 2006). Hwang *et al.* (2008) demonstrated that it showed no genetic toxicity in bacterial reverse mutation and mouse micronucleus assay. The chromosome aberration test using cultured mammalian cells is one of the sensitive methods to predict environmental mutagens and/or carcinogens and is a complementary test to the bacterial reverse mutation test. There is no literature about the genetic toxicity of surfactin C in mammalian cell lines. Therefore, we tested its clastogenicity in cultured mammalian cells, Chinese hamster lung (CHL) cells.

## MATERIALS AND METHODS

***Chemicals.*** Surfactin C (purity > 98%) produced from *Bacillus subtilis* BC1212 was kindly obtained from B&C Biopharm (Suwon, Korea). Cyclophosphamide (Sigma, USA) and mitomycin C (Singm, USA) were served as positive controls. Rat liver S9 induced with Aroclor 1254 (Moltox Inc, USA) was used for metabolic activation.

***Chinese hamster lung (CHL) cell line.*** CHL cells were purchased from the American Type Culture Collection (ATCC, USA) and cultured in Eagle's minimal essential medium (EMEM, Hyclone, USA), supplemented with 10% fetal bovine serum (FBS, Hyclone, USA), in a 37°C incubator containing 5% $CO_2$ without any antibiotics or anti-mycotics. Subcultures were carried out every 2 days, using 0.03% Puck's EDTA and 0.25% Trypsin-EDTA solutions.

***MTT assay.*** Cytotoxicity assay was performed using a two-fold serial dilution gradient of surfactin C concentrations, ranging from 15.6~1000 μg/mL. All treatments were performed in triplicate and the cells were cultured for 18 h in the presence and absence of surfactin C. An $IC_{50}$ concentration was determined from each of the three experiments and the final $IC_{50}$ was determined by averaging the values of the three experiments (± SD). The $IC_{50}$ is defined as the cytotoxicity index that reduces the cell number (by growth inhibition and/or cell killing) to 50% compared with untreated-control CHL cells. The $IC_{50}$ value was used to determine the chromosomal aberration assay concentration ranges.

***Chromosomal aberration assay.*** The assay was performed according to OECD and scientific guidelines (Galloway *et al.*, 1994; OECD, 1997). For the short-period assay with or without S9 mix, CHL cells ($2 \times 10^5$ cells/0.5 ml) were seeded in a 24 well plate culture dish, and incubated in a culture medium for overnight. The surfactin C was added with or without S9 mix and the cultures were incubated for 6 hr. After the 6 hr treatment, the cells were washed with phosphate buffered saline (PBS), and incubated in fresh culture medium for a further 18 h. The continuous treatment test was carried out for 24 h treatment without S9 mix. PBS was used as a negative control, while 20 μg/ml cyclophosphamide (Sigma, USA) and 0.1 μg/ml mitomycin C (Sigma, USA) were used as positive controls for experiments conducted with and without metabolic activation, respectively (Galloway *et al.*, 1994). The cells were treated with colchicine (Sigma, USA; 0.1 μM of final concentration) 2 h before cell harvesting. The cells were trypsinized and incubated in a 75 mM hypotonic KCl solution for 20 min at 37°C. Fixed with acetic acid-ethanol (1 : 3 by volume), and then spread onto clean glass slides. Each slide was stained with 5% Giemsa solution.

Duplicate cultures were used for each experiment. Cells were harvested 24 h after treatment initiation and a minimum of 200 metaphases (100 from each of two duplicate cultures) were analyzed for chromosome damage. Aberrations were classified according to Scott *et al.* (1983) into chromosome and chromatid type damage, with further subdivision into deletions and exchanges. Polyploidy and endoreduplication were recorded as a percentage per 100 metaphases counted.

The frequencies of structural or numerical aberrations were evaluated by the following criteria; less than 5%, 5% to less than 10%, and 10% or more were defined as negative (-), equivocal (±) and positive (+), respectively. The result was considered to be positive if reproducibility was confirmed. Total frequencies of structural aberrations excluded the frequencies of aberrant cells that have gaps only without other aberrations.

***Statistical analysis.*** Statistical analysis was performed using SPSS 12.0k. The differences in the frequency of chromosomal aberrations between groups treated with surfactin C and controls were analyzed by the Fisher's exact test. *P*-values of less than 0.05 were considered to be consistent with statistical significance.

## RESULTS AND DISCUSSION

Genotoxicity is the study of the toxic effects of chemical or physical substances to the gene pool, where

**Table 1.** Summary of results obtained from chromosomal aberration test in CHL cells treated with 6 hr short-treatments of surfactin C

| Compound | Conc. (μg/ml) | S9 mix | Time (hr)[1] | Aberrated cell (%)[2] | |
|---|---|---|---|---|---|
| | | | | -g | +g |
| PBS | 0 | - | 6/18 | 0 | 0 |
| Surfactin C | 125 | - | 6/18 | 2 | 3 |
| | 62.5 | - | 6/18 | 1 | 2 |
| | 31.25 | - | 6/18 | 1 | 1 |
| MMC | 0.1 | - | 6/18 | 42* | 44* |
| PBS | 0 | + | 6/18 | 1 | 1 |
| Surfactin C | 125 | + | 6/18 | 2 | 3 |
| | 62.5 | + | 6/18 | 2 | 2 |
| | 31.25 | + | 6/18 | 1 | 2 |
| CP | 20 | + | 6/18 | 54* | 56* |

MMC, Mitomycin C; CP, Cyclophosphamide.
[1]Treatment time (exposure time/new medium time).
[2]-g, % of cells with chromosome aberrations; +g, % of cells with chromosome aberrations + % of cells with gaps.
*Significantly greater than the corresponding vehicle control, $p < 0.001$.

damages and/or changes to the genetic code or DNA sequence usually occur (Scott *et al.*, 1983). Genotoxic insults may be studied by use of mutagenicity, carcinogenicity and/or teratogenicity assays, due to the close relationship of these three phenomena (Scott *et al.*, 1983; O'Brien *et al.*, 1996; Dearfield *et al.*, 2002).

Chemicals that are not known to cause any chromatogenic aberration must first be tested for the evidence of cytotoxicity in the system chosen for genotoxicity testing (Scott *et al.*, 1983). This would help the establishment of a maximum tolerated dose (MTD) of the chemical being tested, as described above for the mitotic index assay. In our study, the MTT cytotoxicity assay was employed in determining the dose responses of surfactin C directly without any metabolic activation. The $IC_{50}$ value of surfactin C was found 125 μg/ml.

To evaluate the genotoxic activities of surfactin C, we carried out *in vitro* mutagenicity assays. The results obtained in the short-treatment assays for surfactin C with or without S9 mix were shown in Table 1 and 2. In the short-treatments with or without S9 mix, the structural chromosomal aberrations and polyploidy rates were less than 5% for any concentration of the surfactin C. For the continuous treatments test, structural chromosomal aberrations and the polyploidy induction rates were less than 5% at any concentration in both

**Table 3.** Summary of results obtained from chromosomal aberration test in CHL cells treated with 24 hr short-treatments of surfactin C

| Compound | Conc. (μg/ml) | S9 mix | Time (hr)[1] | Aberrated cell (%)[2] | |
|---|---|---|---|---|---|
| | | | | -g | +g |
| PBS | 0 | - | 24 | 1 | 1 |
| Surfactin C | 125 | - | 24 | 2 | 3 |
| | 62.5 | - | 24 | 2 | 3 |
| | 31.25 | - | 24 | 2 | 2 |
| MMC | 0.1 | - | 24 | 43* | 45* |

MMC, Mitomycin C; CP, Cyclophosphamide.
[1]Treatment time (exposure time).
[2]-g, number or % of cells with chromosome aberrations; +g, number or % of cells with chromosome aberrations + number or % of cells with gaps.
*Significantly greater than the corresponding vehicle control, $p < 0.001$.

**Table 2.** Metaphase analysis of chromosomal aberration test in CHL cells treated with 6 hr short-treatments of surfactin C

| Compound | Conc. (μg/ml) | S9 mix | Time (hr)[1] | No. of structural aberration (%)[2] | | | | | | | | | |
|---|---|---|---|---|---|---|---|---|---|---|---|---|---|
| | | | | ctb | csb | cte | cse | Aberration excluding gap (%) | ctg | csg | pol | endo | Aberration including gap (%) |
| PBS | 0 | - | 6/18 | 0 | 0 | 0 | 0 | 0 | 0 | 0 | 0 | 0 | 0 |
| Surfactin C | 125 | - | 6/18 | 2 | 0 | 0 | 0 | 2 | 1 | 0 | 0 | 0 | 3 |
| | 62.5 | - | 6/18 | 1 | 0 | 0 | 0 | 1 | 1 | 0 | 0 | 0 | 2 |
| | 31.25 | - | 6/18 | 1 | 0 | 0 | 0 | 1 | 0 | 0 | 0 | 0 | 1 |
| MMC | 0.1 | - | 6/18 | 20 | 2 | 18 | 2 | 42 | 2 | 0 | 0 | 0 | 44 |
| PBS | 0 | + | 6/18 | 1 | 0 | 0 | 0 | 1 | 0 | 0 | 0 | 0 | 1 |
| Surfactin C | 125 | + | 6/18 | 2 | 0 | 0 | 0 | 2 | 1 | 0 | 0 | 0 | 3 |
| | 62.5 | + | 6/18 | 1 | 0 | 1 | 0 | 2 | 0 | 0 | 0 | 0 | 2 |
| | 31.25 | + | 6/18 | 1 | 0 | 0 | 0 | 1 | 1 | 0 | 0 | 0 | 2 |
| CP | 20 | + | 6/18 | 25 | 5 | 22 | 2 | 54 | 2 | 0 | 0 | 0 | 56 |

MMC, Mitomycin C; CP, Cyclophosphamide.
[1]Treatment time (exposure time/new medium time).
[2]ctb, chromatid break; csb, chromosome break; cte, chromatid exchange; cse, chromosome exchange; ctg, cromatid gap; csg, chromosome gap; pol, polyploidy; endo, endoreduplication.

**Table 4.** Metaphase analysis of chromosomal aberration test in CHL cells treated with 24 hr short-treatments of surfactin C

| Compound | Conc. (μg/ml) | S9 mix | Time (hr)[1)] | No. of structural aberration (%)[2)] | | | | | | | | | |
|---|---|---|---|---|---|---|---|---|---|---|---|---|---|
| | | | | ctb | csb | cte | cse | Aberration excluding gap (%) | ctg | csg | pol | endo | Aberration including gap (%) |
| PBS | 0 | - | 24 | 1 | 0 | 0 | 0 | 1 | 0 | 0 | 0 | 0 | 1 |
| Surfactin C | 125 | - | 24 | 2 | 0 | 0 | 0 | 2 | 1 | 0 | 0 | 0 | 3 |
| | 62.5 | - | 24 | 2 | 0 | 0 | 0 | 2 | 1 | 0 | 0 | 0 | 3 |
| | 31.25 | - | 24 | 1 | 0 | 1 | 0 | 2 | 0 | 0 | 0 | 0 | 2 |
| MMC | 0.1 | - | 24 | 21 | 3 | 18 | 1 | 43 | 2 | 0 | 0 | 0 | 45 |

MMC, Mitomycin C; CP, Cyclophosphamide.
[1]Treatment time (exposure time).
[2]ctb, chromatid break; csb, chromosome break; cte, chromatid exchange; cse, chromosome exchange; ctg, cromatid gap; csg, chromosome gap; pol, polyploidy; endo, endoredupication.

the 24 hr (Table 3 and 4). Based on these data, surfactin C did not induced structural chromosomal aberrations in the short-treatments test and continuous treatment. The results suggested that surfactin C has no clastogenic potential in cultured mammalian cells either with or without S9 activation. This is consistent with the previous reports that surfactin C is negative with or without metabolic activation in mutation assay using *Salmonella typhimurium* and *Escherichia coli* (Hwang *et al.*, 2008).

## References

[CR1] Arima K, Kakinuma A, Tamura G (1968). Surfactin, a crystalline peptidolipid surfactant produced by Bacillus subtilis: isolation, characterization and its inhibition of fibrin clot formation. Biochem. Res. Commun..

[CR2] Banat IM, Makkar RS, Cameotra SS (2000). Potential commercial applications of microbial surfactants. Appl. Microbiol. Biotechnol..

[CR3] Dearfield KL, Cimino MC, McCarroll NE, Mauer I, Valcovic LR (2002). Genotoxicity risk assessment: a proposed classification strategy. Mutat. Res..

[CR4] Desai JD, Banat IM (1997). Microbial production of surfactants and their commercial potential. Microbiol. Mol. Biol. Rev..

[CR5] Galloway S, Aardema M, Ishidate M, Ivett J, Kirkland D, Morita T, Mosesso P, Sofuni T (1994). Report from the working group on in vitro tests for chromosomal aberrations. Mutat. Res..

[CR6] Georgiou G, Liu SC, Sharma MM (1990). Surface active compounds from microorganisms. Biotechnol..

[CR7] Hwang MH, Lim JH, Kim KS, Rhee MH, Kim NW, Kim JC, Park SC (2005). Antibacterial activity in vitro and primary dermal irritationtest in rabbits of surfactin produced in bacillus subtilus complex BC2121. J. Toxicol. Pub. Health.

[CR8] Hwang MH, Lim JH, Yun HI, Rhee MH, Cho JY, Hsu WH, Park SC (2005). Surfactin C inhibits the lipopolysaccharide-induced transcription of interleukin-1beta and inducible nitric oxide synthase and nitric oxide production in murine RAW 264.7 cells. Biotechnol Lett..

[CR9] Hwang YH, Park BK, Lim JH, Kim MS, Song IB, Park SC, Yun HI (2008). Evaluation of genetic and developmental toxicity of surfactin C from Bacillus subtilis BC1212. J. Health Sci..

[CR10] Kanatomo S, Nagai S, Ohki K, Yasuda Y (1995). Study on surfactin, a cyclic depsipeptide. I. Isolation and structure of eight surfuctin analogs produced by *Bacillus natto* KMD 2311. Yakugaku. Zasshi..

[CR11] Kikuchi T, Hasumi K (2002). Enhancement of plasminogen activation surfactin C: augmentation of fibrinolysis in vitro and in vivo. Biochem. Biophys. Acta..

[CR12] Lim JH, Park BK, Kim MS, Hwang MH, Rhee MH, Park SC, Yun HI (2005). The anti-thrombotic activity of surfactins. J. Vet. Sci..

[CR13] Mulligan CN (2005). Environmental applications for biosurfactants. Environ Pollut..

[CR14] O’Brien PJ, Hales BF, Josephy PD, Castonguay A, Yamazoe Y, Guengerich FP (1996). Chemical carcinogenesis, mutagenesis and teratogenesis. Can. J. Physiol. Pharmacol..

[CR15] Organization for Economic CooperationDevelopment OECD (1997). Test Guideline 473, *In vitro* Mammalian chromosome aberration test. OECD Guidelines for the Testing of Chemicals.

[CR16] Park BK, Lim JH, Hwang YH, Kim MS, Song IB, Lee HG, Han SJ, Hwang MH, Kim JW, Park SC, Rhee MH, Yun HI (2006). Acute oral toxicity of surfactin C in mice. J. Toxicol. Pub. Health.

[CR17] Rodrigues L, Banat IM, Teixeira J, Oliveira R (2006). Biosurfactants: potential applications in medicine. J. Antimicrob. Chemother..

[CR18] Singh P, Cameotra SS (2004). Potential applications of microbial surfactants in biomedical sciences. Trends Biotechnol..

[CR19] Scott D, Danford N, Dean B, Kirkland D, Richardson C (1983). *In vitro* chromosome aberration assays. Report of the UKEMS Sub-Committee on Guidelines for Mutagenicity Testing, Part 1.

[CR20] Takahashi T, Ohno O, Ikeda Y, Sawa R, Homma Y, Igarashi M, Umezawa K (2006). Inhibition of lipopolysaccharide activity by a bacterial cyclic lipopeptide surfactin. J. Antibiot (Tokyo).

